# Supplementary figures and images for: Transcriptomic profiling of epigenetic regulators and metabolic reprogramming in human cholangiocarcinoma
Source: Front Cell Dev Biol. 2026 Jun 4;14:1765945. doi: 10.3389/fcell.2026.1765945 (PMC13275250; doi:10.3389/fcell.2026.1765945)

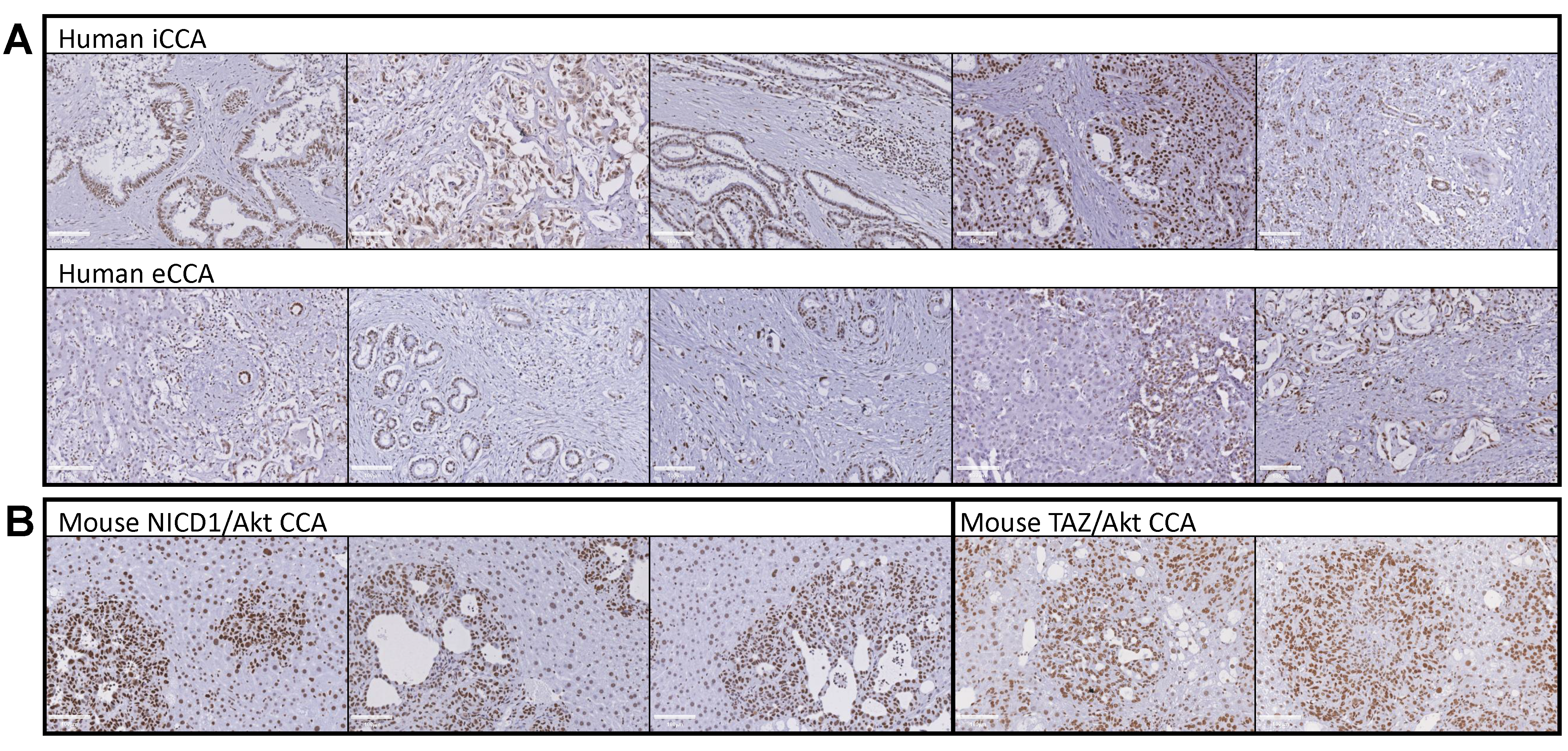

Supplement: Supplementary file 1 [file Image3.tiff]

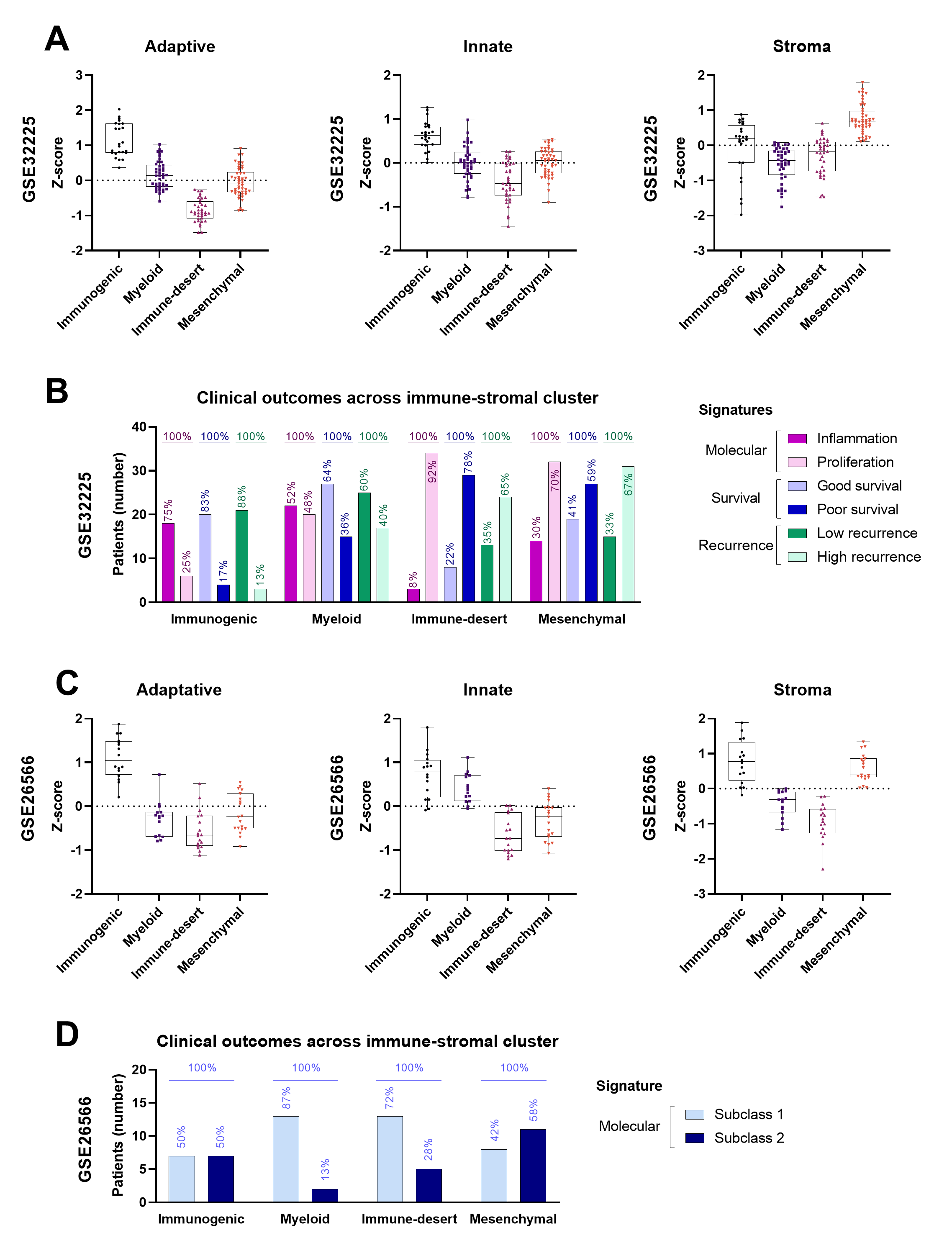

Supplement: Supplementary file 6 [file Image2.tiff]
