# Supplementary material for: Transcriptomic profiling of epigenetic regulators and metabolic reprogramming in human cholangiocarcinoma
Source: Front Cell Dev Biol. 2026 Jun 4;14:1765945. doi: 10.3389/fcell.2026.1765945 (PMC13275250; doi:10.3389/fcell.2026.1765945)
Supplement: Supplementary file 3 [file Supplementaryfile1.docx]

Supplementary Material

# Supplementary Figures and Tables

**Supplementary Table 1.** List of EpiGs selected from the literature, classified by their epigenetic function and family, and manually curated functional activities.

**Supplementary Table 2.** List of MGs functionally linked to epigenetic regulation, selected from the literature and classified according to the biological process in which they participate.

**Supplementary Table 3.** List of KEGG metabolic pathways analyzed.

**Supplementary Table 4.** List of RLEs retrieved from RLEdb, and manually curated EC numbers, associated metabolic pathways, and functional classifications.

**Supplementary Table 5.** iCCA patients' classification by transcriptomic signatures into the survival- and recurrence-related subclasses in the GSE32225.

**Supplementary Table 6.** CCA patients' classification by transcriptomic signatures into the subclasses 1 and 2 and the Hsiao liver-specific subclasses in the GSE26566.

**Supplementary Table 7.** GO, KEGG, and Hallmark gene sets in GSEA analysis of DEGs between CCA and NBD in GSE32225 (Sia et al., 2013), GSE26566 (Andersen et al., 2012), and GSE132305 (Montal et al., 2020). Enriched terms (over- or underrepresented) with statistically significant NES (adjusted *p*<0.05) that included EpiGs among the DEGs were highlighted (≥10 highlighted in orange and ≥3 EpiGs in yellow).

**Supplementary Table 8.** Average expression (Log_2_FC) of EpiGs, MGs and RLEs across GSE32225 (Sia et al., 2013), GSE26566 (Andersen et al., 2012), and GSE132305 (Montal et al., 2020) human datasets comparing CCA and NBD samples, human CCA tumoroids and healthy liver-derived organoids (Broutier et al., 2017), and TAZ/Akt-driven tumors and NICD1/Akt-driven tumors (early to advanced stages) compared to normal mouse livers.

**Supplementary Table 9.** GO, KEGG, and Hallmark gene sets in GSEA analysis of DEGs in CCA samples, comparing the worst- versus best-prognosis groups across the GSE32225 (Sia et al., 2013) and GSE26566 (Andersen et al., 2012) cohorts. Enriched terms (over- or underrepresented) with statistically significant NES (adjusted *p*<0.05) that included EpiGs among the DEGs were highlighted (≥10 highlighted in orange and ≥3 EpiGs in yellow).

**Supplementary Table 10.** Differential expression of EpiGs, MGs and RLEs across TME- defined CCA immune-stromal clusters. Tumors were grouped into Immunogenic, Myeloid, Immune Desert, and Mesenchymal patterns using MCP-counter deconvolution of bulk transcriptomic data. Differentially expressed genes (raw p<0.05) were identified in GSE32225 (Sia et al., 2013) and Hsiao liver-specific subclasses in the GSE26566.

## Supplementary Figures


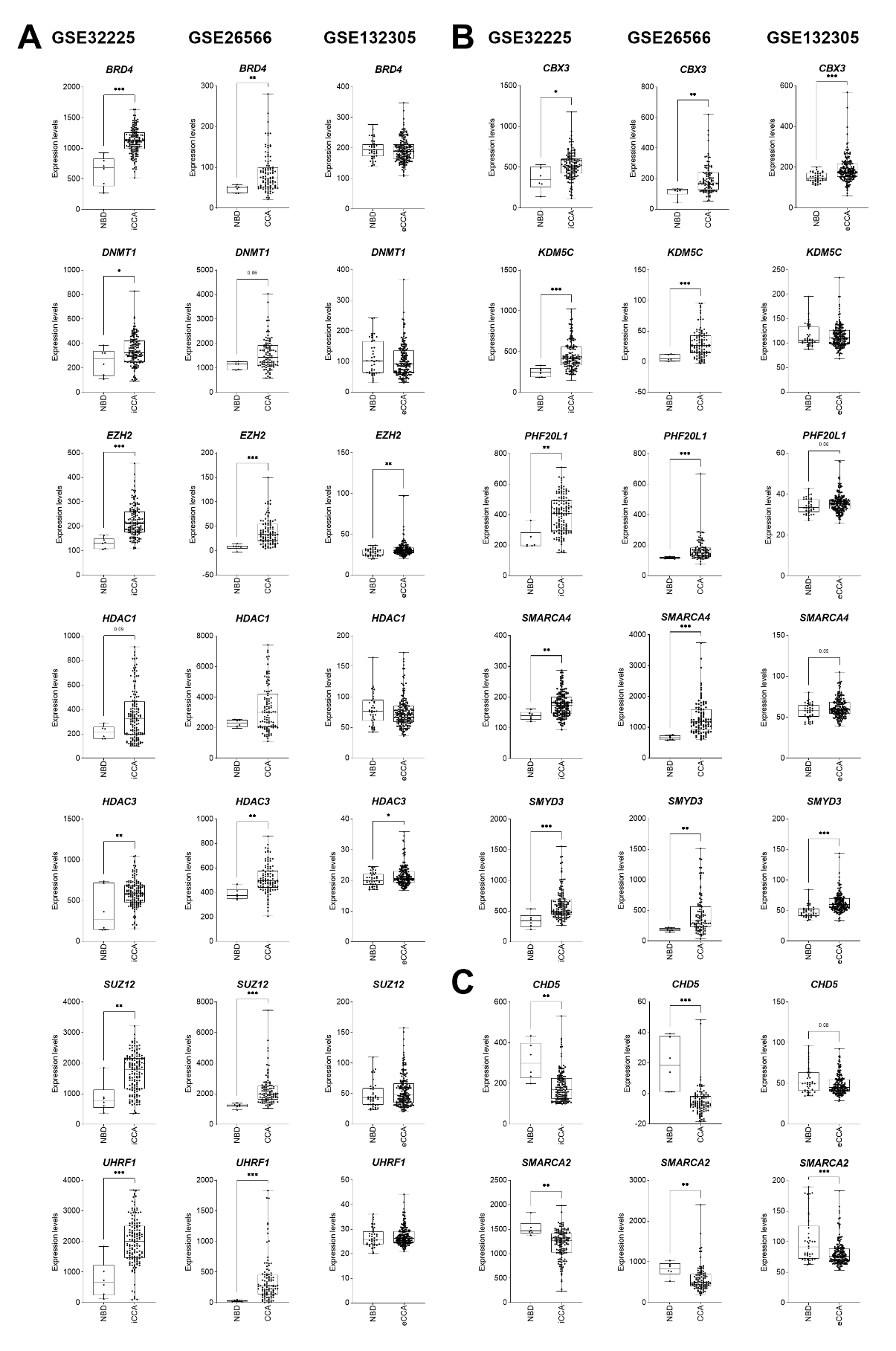
**Supplementary Figure 1. Expression of selected EpiGs in human CCA.** **(A)** EpiGs reported as overexpressed in CCA. **(B)** Additional upregulated and **(C)** downregulated EpiGs with limited or no prior evidence in CCA, representing potentially novel candidates.



**Supplementary Figure 2. Shared and divergent pathway enrichment and gene expression in human iCCA and eCCA.** **(A)** Subset of pathways consistently altered in CCA compared with NBD samples across GSE32225, GSE26566, and GSE132305 human datasets. **(B)** Pathways showing opposite enrichment directions between iCCA (GSE32225) and eCCA (GSE132305) compared with NBD samples. Overlapping differentially expressed genes (DEGs) across all comparisons, highlighting a subset of consistently **(C)** upregulated and **(D)** downregulated genes, including EpiGs, MGs, and RLEs, reflecting shared transcriptional programs in CCA.

**
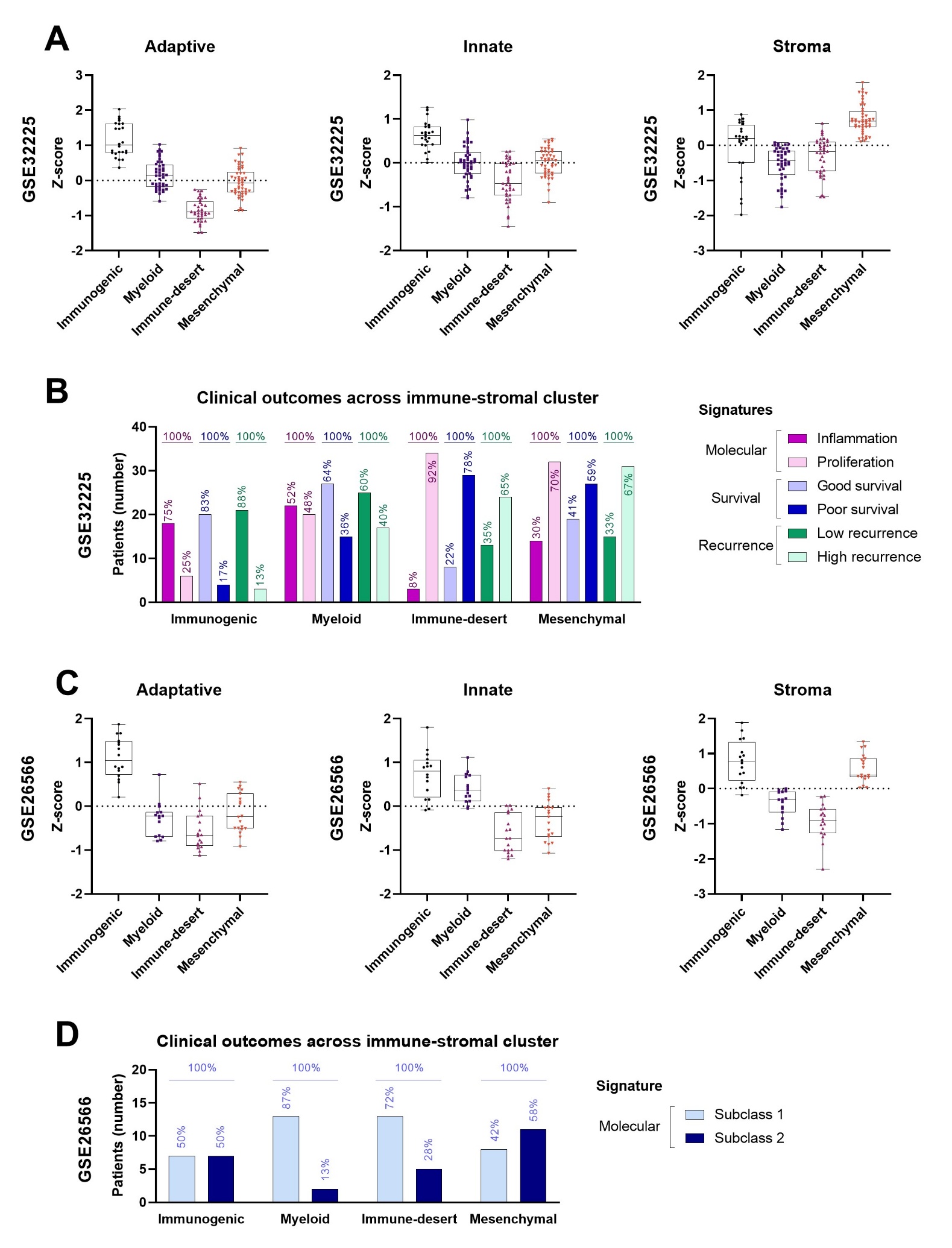
**

**Supplementary Figure 3. Immune-stromal landscape and clinical subclass distribution across CCA clusters.** **(A)** Distribution of immune-stromal composite axes across CCA clusters in GSE32225. Boxplots showing the distribution of the three composite axes (adaptive immunity, innate immunity, and stromal activation) across the four immune-stromal clusters (Immunogenic, Myeloid-rich, Immune-desert, and Mesenchymal). Axes were calculated as the median of standardized MCP-counter scores (Z-scores present relative enrichment across samples for each cell type). **(B)** Distribution of clinical subclasses across clusters in GSE32225. Bar plots display the proportion of patients in each cluster across clinical subclasses (inflammation/proliferation, survival, and recurrence), illustrating the distribution of low- and high-aggressiveness groups. **(C)** Immune-stromal composite axis distribution in Hsiao_High patients from GSE26566. Boxplots depict adaptive, innate, and stromal scores across the four clusters, derived from median standardized MCP-counter values. **(B)** Clinical subclass composition across clusters in Hsiao_High patients from GSE26566. Bar plots show the distribution of patients within each cluster by inflammation/proliferation status, survival, and recurrence.


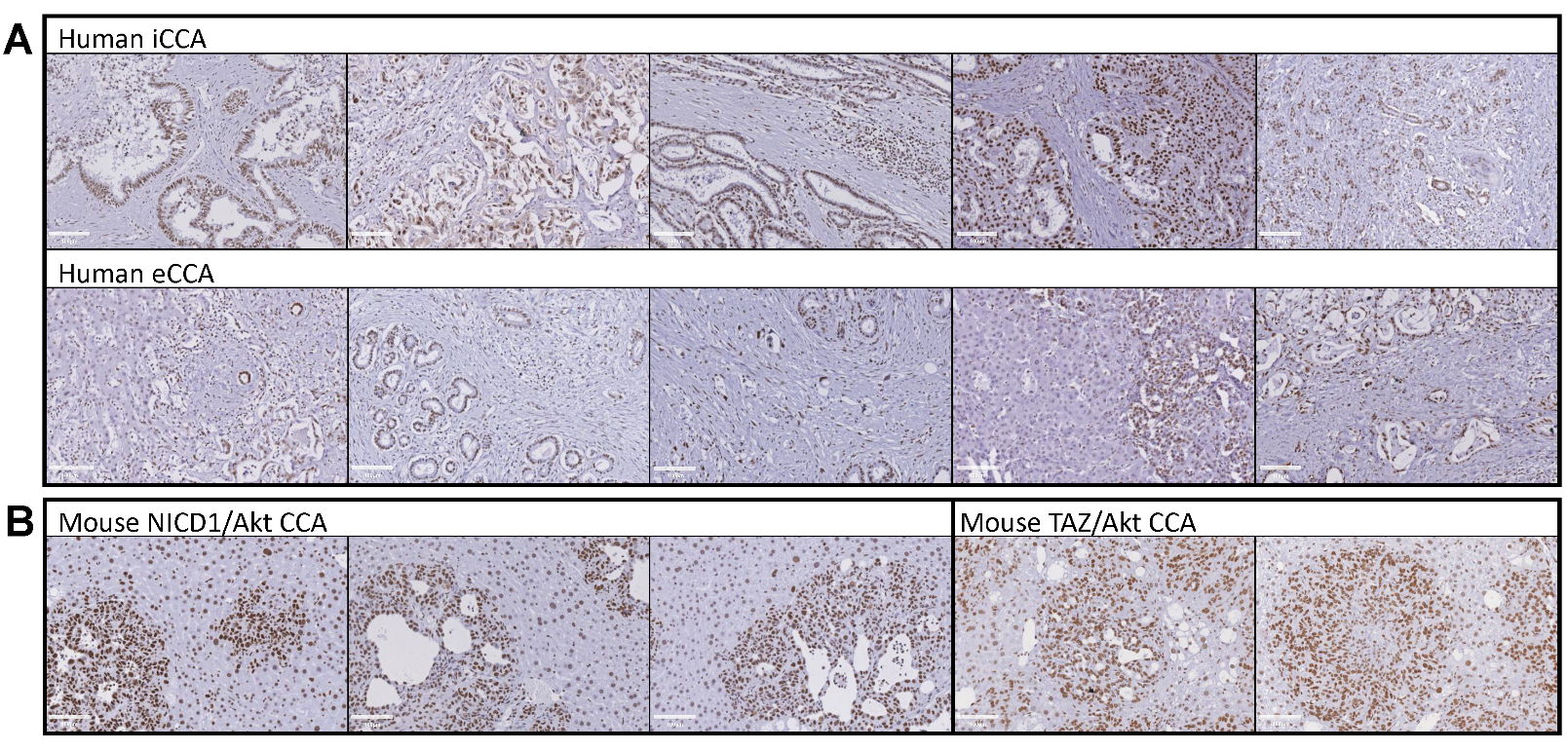


**Supplementary Figure 4. SMARCA4 protein expression in human and experimental CCA. (A)** Representative SMARCA4 immunostaining in human iCCA and eCCA samples and in **(B)** TAZ/Akt and NICD1/Akt mouse CCA models. Scale bar: 100 µm.
