# Supplementary material for: Transcriptomic profiling of epigenetic regulators and metabolic reprogramming in human cholangiocarcinoma
Source: Front Cell Dev Biol. 2026 Jun 4;14:1765945. doi: 10.3389/fcell.2026.1765945 (PMC13275250; doi:10.3389/fcell.2026.1765945)
Supplement: Supplementary file 4 [file DataSheet1.pdf]

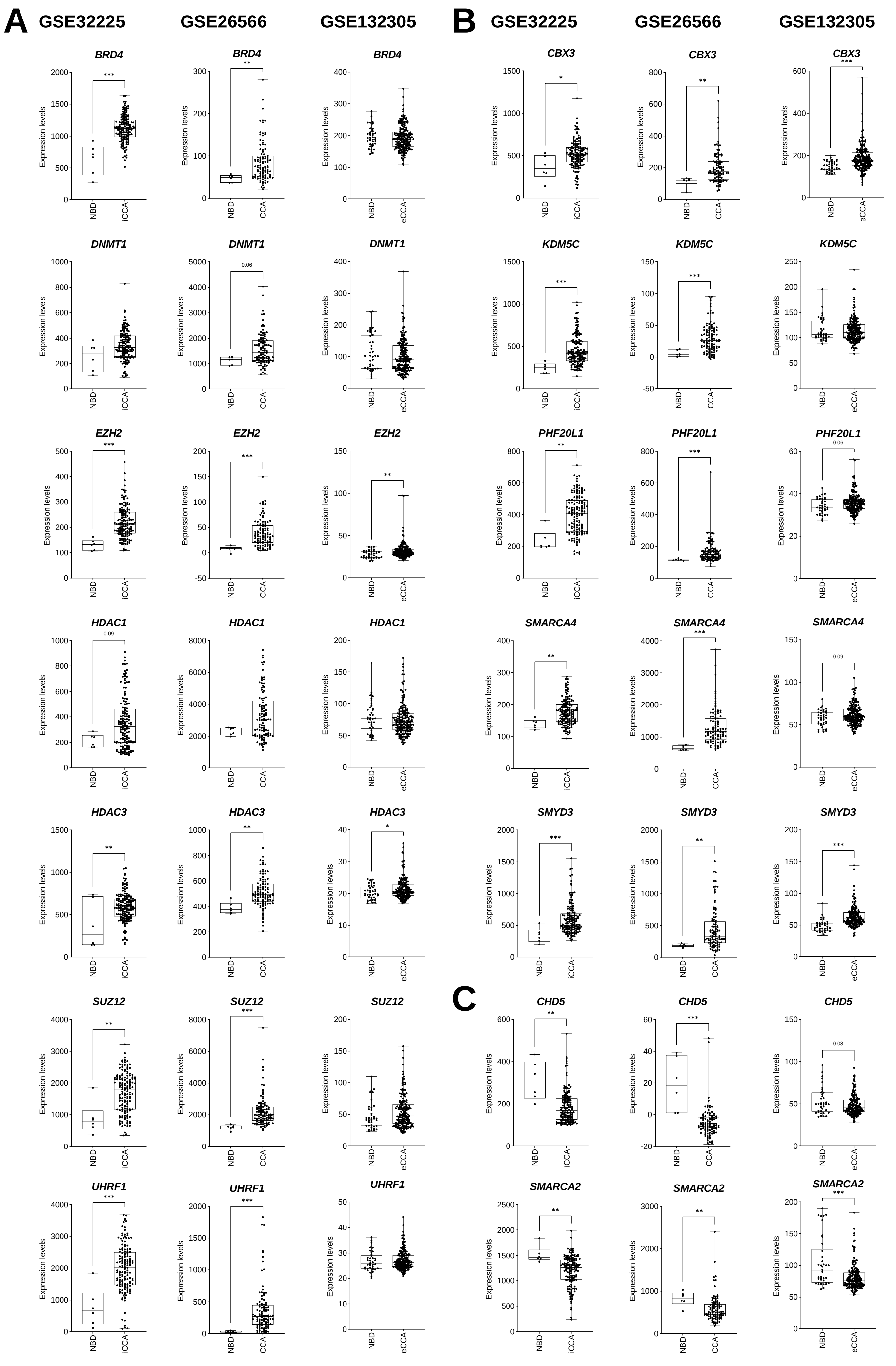

A
GSE32225
GSE26566
GSE132305
B
GSE32225
GSE26566
GSE132305
BRD4
***
Expression levels
NBD
iCCA
BRD4
**
NBD
CCA
BRD4
NBD
eCCA
CBX3
*
CBX3
**
CBX3
***
DNMT1
DNMT1
0.06
DNMT1
KDM5C
***
KDM5C
***
KDM5C
EZH2
***
EZH2
***
EZH2
**
PHF20L1
**
PHF20L1
***
PHF20L1
0.06
HDAC1
0.09
HDAC1
HDAC1
SMARCA4
**
SMARCA4
***
SMARCA4
0.09
HDAC3
**
HDAC3
**
HDAC3
*
SMYD3
***
SMYD3
**
SMYD3
***
C
SUZ12
**
SUZ12
***
SUZ12
CHD5
**
CHD5
***
CHD5
0.08
UHRF1
***
UHRF1
***
UHRF1
SMARCA2
**
SMARCA2
**
SMARCA2
***
